# Early transmission patterns of coronavirus disease 2019 (COVID-19) in travellers from Wuhan to Thailand, January 2020

**DOI:** 10.2807/1560-7917.ES.2020.25.8.2000097

**Published:** 2020-02-27

**Authors:** Pilailuk Okada, Rome Buathong, Siripaporn Phuygun, Thanutsapa Thanadachakul, Sittiporn Parnmen, Warawan Wongboot, Sunthareeya Waicharoen, Supaporn Wacharapluesadee, Sumonmal Uttayamakul, Apichart Vachiraphan, Malinee Chittaganpitch, Nanthawan Mekha, Noppavan Janejai, Sopon Iamsirithaworn, Raphael TC Lee, Sebastian Maurer-Stroh

**Affiliations:** 1 Department of Medical Sciences, Ministry of Public Health, Thailand; 2 Department of Disease Control, Ministry of Public Health, Thailand; 3 Thai Red Cross Emerging Infectious Diseases - Health Science Centre, Chulalongkorn University, Thailand; 4 Bioinformatics Institute, Agency for Science Technology and Research, Singapore; 5 Department of Biological Sciences, National University of Singapore, Singapore

**Keywords:** Wuhan, coronavirus, traveller, transmission, COVID-19, SARS-CoV-2

## Abstract

We report two cases of coronavirus disease 2019 (COVID-19) in travellers from Wuhan, China to Thailand. Both were independent introductions on separate flights, discovered with thermoscanners and confirmed with RT-PCR and genome sequencing. Both cases do not seem directly linked to the Huanan Seafood Market in Hubei but the viral genomes are identical to four other sequences from Wuhan, suggesting early spread within the city already in the first week of January.

In late December 2019, an outbreak with an initially undiagnosed pneumonia was reported in the city of Wuhan, Hubei Province, China, and linked to the Huanan Seafood Market [1,2]. The causative pathogen was identified as a novel betacoronavirus within the severe acute respiratory syndrome (SARS) coronavirus (CoV) family, recently termed SARS-CoV-2 [3-7]. In response to the outbreak, several countries including Thailand, established thermal screening at the airport for travellers from Wuhan since 3 January. On 8 January and 13 January, suspected cases of infection with SARS-CoV-2 were identified at Bangkok Suvarnabhumi airport. We report the investigation, basic clinical characteristics and viral genomes derived from these cases.

## Case 1

A woman in her early 60s from Wuhan developed a fever with chills, sore throat and headache on 5 January 2020. She went to a local health facility in China and received undisclosed medication. On 8 January 2020, she took a direct ca 4 h flight to Thailand from Wuhan, with five family members, as part of a tour group of 16 (including the case). Her measured temperature at the arrival gate was 38.6 °C by thermoscanner, and confirmed with a tympanic thermometer. After being interviewed by quarantine officers, she was transferred to Bamrasnaradura Infectious Disease Institute (BIDI) Hospital, Nonthaburi, for isolation and laboratory investigations. She reported a runny nose and sore throat but no dyspnea or diarrhoea. Upon admission, her vital signs were normal except for elevated blood pressure. Her physical examination was unremarkable including inconspicuous lung sounds. Her complete blood count suggested a viral infection from relatively decreased neutrophil (48%; norm: 35–75%) to lymphocyte (40%; norm: 20–59%) ratio [8]. The chest X-ray (CXR) results on 8 January were compatible with pneumonia with mild thickening lung marking at both lower lungs possibly because of crowded lung rather than infiltration. It also showed borderline cardiomegaly. Repeat CXR after 7 days showed no remarkable changes.

In the interview, the patient explicitly stated that she had not visited the Huanan Seafood Market but she was living near another local market, which she visited daily until illness onset. This market was at ca 7.5 km distance from the Huanan Seafood Market. The patient also reported that she had not purchased live animals and or visited stalls with live animals. She did not visit Jinyintai Hospital or other hospitals in Wuhan. However, she visited local dispensaries in Wuhan to obtain medication. She also reported no contact with persons with respiratory symptoms.

Clinical specimens collected on admission included the upper respiratory tract secretions and sputum. These specimens tested positive on 12 January for the CoV family by using a conventional nested RT-PCR [9]. Genomic sequencing analysis included Sanger and next generation sequencing were performed by the Emerging Infectious Diseases Health Science Center, the Thai Red Cross Society and the Thai National Institute of Health, Department of Medical Sciences and the sequence shared via the Global Initiative on Sharing All Influenza Data (GISAID) EpiCoV database (EPI_ISL_403962). The sequencing protocol and details are provided in the Supplementary Materials. The patient recovered after testing negative for SARS-CoV-2, and returned to China without signs and symptoms on 18 January 2020.

## Case 2

A woman in her mid-70s from Wuhan landed at Suvarnabhumi airport on 13 January. She travelled to Thailand with three family members as part of a tour group of 20 (including the case). An airport thermoscanner detected a fever of 38.0 °C that was confirmed with a tympanic thermometer. The patient reported a sore throat, that her fever onset date was 13 January and that she had a cough for an undetermined period. The patient was hospitalised at BIDI. Upon admission, she reported mild tachypnoea, and her CXR was compatible with pneumonia. Similar to case 1, the first CXR taken on 13 January, showed thickening interstitial lung marking at both lower lung fields and both perihilar regions because of interstitial infiltration or crowded lung marking, mild cardiomegaly and dilated aorta. Follow-up CXR on 17 January additionally showed recent hazy with reticular opacities at left middle lung field. The patient was not in severe condition but stable.

In the patient interview, she reported that she did not visit the Huanan Seafood Market or other markets. She also reported no contact with pigs, camels, other mammals (or areas with dead birds), or any consumption of raw or undercooked foods. She stated that she was not in contact with persons with respiratory symptoms.

A conventional nested RT-PCR test of this patient was positive for the CoV family [9]. Subsequent genome sequencing was again compatible with the SARS-CoV-2 and shared via the GISAID EpiCoV database (EPI_ISL_403963). A nasopharyngeal swab also tested positive by RT-PCR for adenovirus. The patient was no longer febrile as of 17 January, and after testing negative for the CoV family by conventional nested RT-PCR, she was discharged and she returned to China.

## Genome sequence analysis

**Figure 1:**
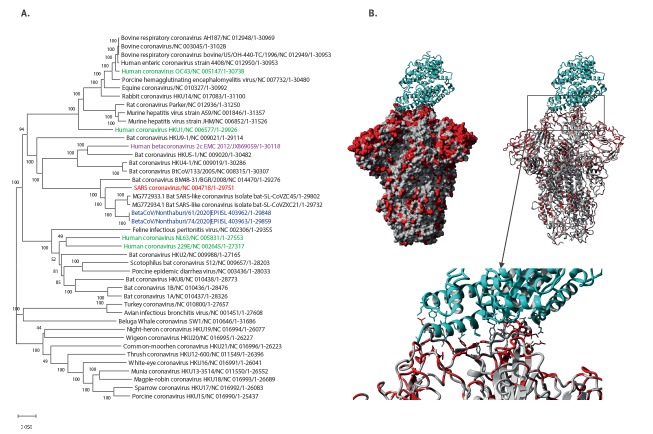
Phylogenetic trees of Thai sequences in context of all coronavirus families (A) and structural mapping of mutations in the spike glycoprotein between SARS CoV (PDB:6CG [12]) and the current SARS-CoV-2 using YASARA [20] (B)

CoV: coronavirus; MERS: Middle East respiratory syndrome coronavirus; SARS: severe acute respiratory syndrome.

Comparing the two genome sequences with a non-redundant selection of representatives from all known CoV families by alignment [10] and phylogenetic tree (Figure 1A) [11] shows that they belong to the SARS family of betacoronaviruses and while related to SARS-CoV (80% genome identity), they were most closely related to SARS-like bat CoV from China (88% identity) as closest known sequence at the time of emergence.

Panel A: blue: SARS-CoV-2; red: SARS; purple: MERS; green: common cold.

Panel B: cyan: ACE2 human host receptor; grey: CoV spike glycoprotein trimer (PDB:6ACG); red: mutations between SARS-CoV vs SARS-CoV-2.

The phylogenetic tree was created from whole genome alignment with MAFFT using the neighbour-joining method with maximum composite likelihood (MCL) model, uniform site rates and 500 bootstrap tests using MEGA X.

Structural mapping of mutations in the spike glycoprotein between SARS CoV and the two cases of the SARS-CoV-2 reported here shows only 76% identity at the protein level (Figure 1B). This surface protein is critical for ACE2 host receptor interaction and is also a target of the immune response [12,13]. Given several mutations in the binding interface, it may differ in host cell binding efficiency compared with SARS-CoV which could result in differences in virulence and transmission potential [14,15].

**Figure 2:**
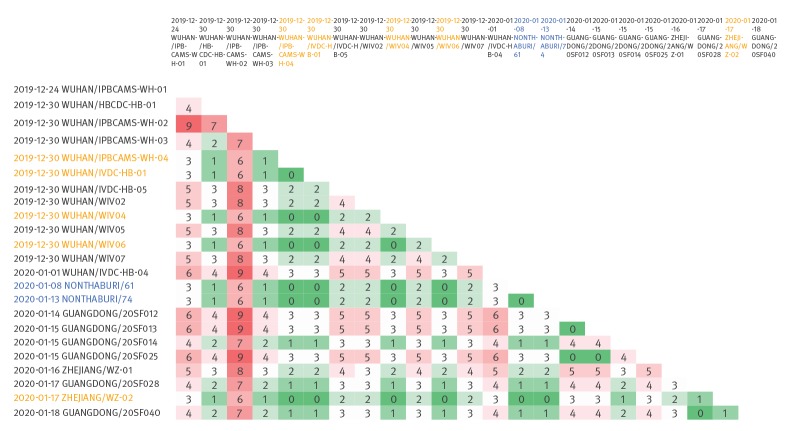
Within-outbreak SARS-CoV-2 sequence divergence and clusters, China and Thailand, January 2020

The genomes of the two separate cases of coronavirus disease 2019 (COVID-19) are identical over the full length of close to 30 kb and are furthermore identical to five other sequences (four from Wuhan and one from Zhejiang); together these sequences form the largest cluster of identical cases within the early outbreak, comprising a core of at least indirectly linked cases (Figure 2). Within-outbreak sequence divergence is generally low with 0–9 nt differences over the whole genome and mutations unique to individual strains are possibly related to quality differences of the samples and noise of the methods used for sequencing.

Number of pair-wise nt differences across whole genomes colour-coded from zero (green) to nine (red). Blue: Thai sample names. Orange: samples with sequences identical to each other and the Thai sequences.

We gratefully acknowledge the authors, the originating and submitting laboratories for their sequence and metadata shared through GISAID, on which this research is based (as listed in Supplementary Table 1).

## Follow-up of contacts

Case 1 travelled in a tour group with 15 others and 40 close contacts were identified: 15 members of the tour group, 15 people sitting within two rows in front and back of the seat of case 1 on the same airplane, nine crew members and one port health officer. They were monitored for COVID-19 with RT-PCR tests on days 1, 12 and 14 ; all tested negative.

Case 2 travelled in a tour group with 19 others and 44 close contacts were identified: 19 members of the tour group, 15 within two rows front and back of the seat of case 2, nine crew members and one port health officer. All were monitored for COVID-19 with RT-PCR tests on days 1, 12 and 14 and all tested negative.

## Discussion and conclusion

According to the cases’ history, the two imported COVID-19 cases described here are not directly linked, yet their genomes are identical. Further, according to information provided by the cases, they have no direct link to the Huanan Seafood Market but their genomes are identical to four sequences from Wuhan collected on 30 December 2019 and sequenced by three different laboratories, indicating potential wider distribution in the city. Although within-outbreak sequence divergence is low for this virus, identical genomes of up to 30 kb are rare and a strong sign of recent transmission linkage, albeit with unknown number of generations within the transmission chain. The missing link to the market, the time of travel and onset of symptoms in the two COVID-19 cases together with the incubation period of mean 6.4 days (range: 5.6–7.7) [16] and the time of closest genome neighbours obtained from sequences in Wuhan, suggest that transmission within Wuhan beyond the Huanan Seafood Market is likely to have occurred in the first week of January or earlier. People travelling out of the city since then may have spread the virus further before travel restrictions were enforced on 23 January [17].

Thailand implemented measures for screening patients travelling from Wuhan since 3 January 2020 at Suvarnabhumi Airport, Don Mueang, Phuket and Chiang Mai airports, and stepped up surveillance at public and private hospitals. The two cases described here who tested positive for SARS-CoV-2 were the first confirmed exported cases from China, suggesting early international spread. Therefore, while one cannot exclude the possibility that asymptomatic cases in their incubation period would be missed and become infectious later, screening of incoming travellers from an affected area proved successful at least in these two instances, when COVID-19 cases were detected, isolated, observed and only discharged once they tested negative for SARS-CoV-2. Rapid response including genome sequencing and sharing via GISAID [18,19] (https://www.gisaid.org/) enabled fast dissemination of results and provided a glimpse of early transmission patterns in this outbreak.

## Supplementary Data

137000 Supplement S1

## Supplementary Data

43000 Supplementary Table S1
