# Supplementary material for: Early transmission patterns of coronavirus disease 2019 (COVID-19) in travellers from Wuhan to Thailand, January 2020
Source: Euro Surveill. 2020 Feb 27;25(8):2000097. doi: 10.2807/1560-7917.ES.2020.25.8.2000097 (PMC7055038; doi:10.2807/1560-7917.ES.2020.25.8.2000097)
Supplement: Supplement S1 [file 20-00097_OKADA_SupplementS1.pdf]

Supplement S1: Methods description for sequencing, phylogenetic and structural analysis

This supplementary material is hosted by *Eurosurveillance* as supporting information alongside the article “Early transmission patterns of coronavirus disease 2019 (COVID-19) in travellers from Wuhan to Thailand, January 2020”, on behalf of the authors, who remain responsible for the accuracy and appropriateness of the content. The same standards for ethics, copyright, attributions and permissions as for the article apply. Supplements are not edited by *Eurosurveillance* and the journal is not responsible for the maintenance of any links or email addresses provided therein.

Illumina Miseq Procedures

First strand synthesis reaction buffer and random primer mix (2X) were prepared by mixing 5 µl of purified mRNA (10-100 ng) with 4 µlof NEBNext first strand synthesis reaction buffer (5X), 1 µl of random primers in a nuclease-free tube. The tube is then incubated at 94°C for 15 minutes and placed on ice. The first strand cDNA synthesis was conducted by mixing 10 µl of primed mRNA with 0.5 µl of Murine RNase Inhibitor, 1 µl of ProtoScript II Reverse Transcriptase, 8.5 µl of nuclease-free water. Tube was then incubated in a preheated thermal cycler with the following cycler condition: 25 ˚C for 10 minutes, 42 ˚C for 50 minutes, 70 ˚C for 15 minutes, and hold at 4 ˚C. Second strand cDNA synthesis was conducted immediately after this step by mixing 20 µl of first strand synthesis reaction with 8 µl of Second Strand Synthesis Reaction Buffer (10X), 4 µl of Second Strand Synthesis Enzyme Mix, and 48 µl of nuclease-free water. The tube was thoroughly mixed by gentle pipetting and incubated in a thermal cycler at 16 ˚C for 1 hour, with heated lid set at 40 ˚C.

To purify double-stranded cDNA, vortex AMPure XP beads to resuspend, and then ddd 144 µl (1.8X) of resuspended AMPure XP beads to the second strand synthesis reaction (~80 µl), mix well on a vortex mixer or by pipetting up and down at least 10 times. The tube was then incubated for 5 minutes at room temperature, quickly spun in a microcentrifuge to collect any sample on the sides of the tube, and placed on an appropriate magnetic rack to separate beads from supernatant. After the solution was clear (about 5 minutes), the supernatant was carefully removed and discarded, avoiding any disturbing the beads that contain DNA targets. While on magnetic rack, the beads were washed (2 times) by adding 200 µl of freshly prepared 80% ethanol to the tube, incubated at room temperature for 30 seconds, supernatant was then carefully removed and discarded. The beads were subsequently air dried for 5 minutes while the tube was on the magnetic rack with lid open. Once dried, the tube was removed from the magnet rack, DNA target was eluted from the beads by adding 60 µl 0.1X TE Buffer or 10 mM Tris-HCl pH 8.0. Tube was mixed on a vortex mixer or by pipetting up and down, quickly spun and incubated for 2 minutes at room temperature, and placed on magnetic rack. Once the solution was clear, 55.5 µl of the supernatant was transferred to a clean nuclease-free PCR tube.

To perform end prep of cDNA library, 55.5 µl of the supernatant in a sterile nuclease-free tube was mixed with 6.5 µl NEBNext End Repair Reaction Buffer (10X) and 3 µl of NEBNext End Prep Enzyme Mix, incubated in a preheated thermal cycler with lid set at 75 °C using the following cycler condition: 20 ˚C for 30 minutes, 65 ˚C for 30 minutes, 70 ˚C for 15 minutes, and hold at 4 ˚C. Adaptor ligation was performed

immediately by diluting NEBNext Adaptor for Illumina (15 μM) to 1.5 μM with a 10-fold dilution (1:9) with 10 mM Tris-HCl, or 10 mM Tris-HCl, with 10 mM NaCl for immediate use. The dA-tailed cDNA (65 μl) was then mixed with 15 μl of Blunt/TA Ligase Master Mix, 1 μl of NEBNext Adaptor (1.5 μM), and 2.5 μl of nuclease-free water, mixed by pipetting, spun to collect all liquid from the sides of the tube, and incubated for 15 minutes at 20 °C in a thermal cycler with heated lid on the thermal cycler turned off. The ligation mixture was then mixed with 3 μl of USER Enzyme, mixed, and incubated at 37 °C for 15 minutes.

To purify the ligation reaction, the ligation reaction was mixed with nuclease-free water to to bring the reaction volume to 100 μl, and then added with 100 μl (1.0X) resuspended AMPure XP Beads, mixed on a vortex mixer or by pipetting up and down at least 10 times. The reaction was subsequently incubated for 5 minutes at room temperature, quickly spun in a microcentrifuge, and placed on an appropriate magnetic rack to separate beads from the supernatant. After the solution was clear (about 5 minutes), supernatant containing unwanted fragments was discarded (caution: do not discard the beads). While on magnetic rack, the beads were washed (2 times) by adding 200 μl of freshly prepared 80% ethanol to the tube, incubated at room temperature for 30 seconds, supernatant was then carefully removed and discarded. The tube was spun and put back in the magnetic rack, residual ethanol was removed and the beads were air dried for 5 minutes while the tube was on the magnetic rack with the lid open. Once dried, DNA target from the beads was eluted with 52 μl 0.1X TE or 10 mM Tris-HCl. Tube was mixed on a vortex mixer or by pipetting up and down, quickly spun and incubated for 2 minutes at room temperature, and placed on magnetic rack. Once the solution was clear, 50 μl of the supernatant was transferred to a clean nuclease-free PCR tube, added with 50 μl (1.0X) of the resuspended AMPure XP Beads, mixed well on a vortex mixer or by pipetting up and down at least 10 times, and incubated for 5 minutes at room temperature. The tube was subsequently spun in a microcentrifuge and placed on an appropriate magnetic rack to separate beads from the supernatant. After the solution was clear (about 5 minutes), supernatant that contained unwanted fragments was discarded (caution, do not disturb the beads). A washing step (2 times) was performed by adding 200 μl of freshly prepared 80% ethanol to the tube while in the magnetic rack and incubated at room temperature for 30 seconds, supernatant was then carefully removed and discarded. The tube was spun and put back in the magnetic rack, residual ethanol was removed and the beads were air dried for 5 minutes while the tube was on the magnetic rack with the lid open. Once dried, DNA target from the beads was eluted with 22 μl 0.1X TE or 10 mM Tris-HCl. Tube was mixed on a vortex mixer or by pipetting up and down, quickly spun and incubated for 2 minutes at room temperature, and placed on magnetic rack. Without disturbing the bead pellet, 20 μl of the supernatant was transferred to a clean PCR tube.

A PCR enrichment of adaptor ligated DNA process was performed by mixing 23 μl of cDNA with 25 μl of NEBNext High-Fidelity PCR Master Mix (2X), 1 μl of Universal PCR Primer (25 μM), and 1 μl of Index (X) Primer (25 μM). Tube was then incubated in a thermal cycler with the following cycler condition: 1 cycle of initial denaturation (98 ˚C for 30 seconds); 12-15 cycles of denaturation (98 ˚C for 10 seconds), annealing (65 ˚C for 30 seconds), and extension (72 ˚C, 30 seconds); and 1 cycle of final extension (72 ˚C for 5 minutes, and hold at 4 ˚C.

PCR reaction was purified by adding 50 µl of the PCR reaction with 45 µl suspended Agencourt AMPure XP Beads. The mixer was mixed on a vortex mixer or by pipetting up and down at least 10 times, incubated for 5 minutes at room temperature, spun in a microcentrifuge, and placed on an appropriate magnetic rack to separate beads from the supernatant. After the solution was clear (about 5 minutes), supernatant was carefully removed and discarded. A washing step (2 times) was performed by adding 200 µl of freshly prepared 80% ethanol to the tube while in the magnetic rack and incubated at room temperature for 30 seconds, supernatant was then carefully removed and discarded. The beads were air dried for 5 minutes while the tube was on the magnetic rack with the lid open. Once dried, DNA target from the beads was eluted with 23 µl 0.1X TE. Tube was mixed on a vortex mixer or by pipetting up and down, quickly spun and incubated for 2 minutes at room temperature, and placed on magnetic rack. Without disturbing the bead pellet, 20 µl of the supernatant was transferred to a clean PCR tube. Libraries could be stored at –20 °C if not used immediately.

Figure: Recommended size selection conditions for libraries with insert sizes > 300 bp.

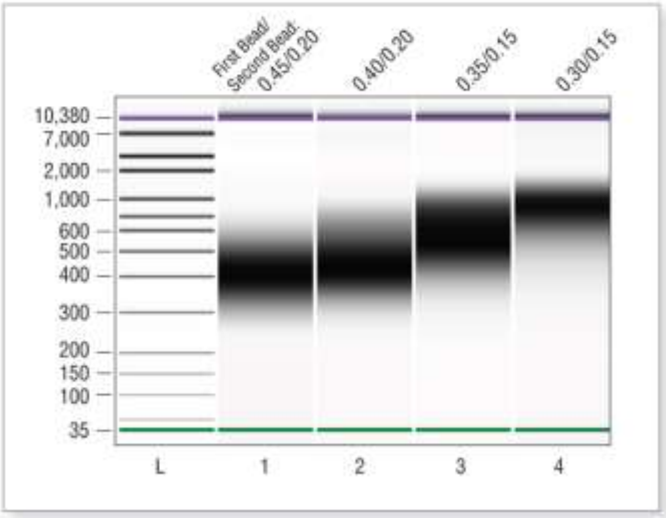

Initial Bioinformatics analysis

Using the protocol described above we have sequenced 100% of the virus from the patients and made available on GISAID (https://www.gisaid.org) [1,2] under accessions EPI_ISL_403962 and EPI_ISL_403963. In assembling the read data from the sputum specimen to the Wuhan Bat CoV virus reference sequence, the coverage and read depth was similar to that from the upper respiratory specimen. To obtain a complete consensus of the virus, the sequencing reads from the sputum and nasopharyngeal specimens from this patient were pooled. Consensus sequences were generated from threshold coverage values of 2 and 4.

Phylogenetic analysis

Phylogenetic tree of Wuhan CoV full genome sequences in context of representatives of all CoV families (alignment with MAFFT [3] whole genome Neighbor Joining, Maximum Composite Likelihood, uniform rates, 500 bootstrap, using MEGA X [4]).

Estimates of Evolutionary Divergence between Sequences in Figure 2: The number of base differences per sequence from between sequences are shown. This analysis involved 23 nucleotide sequences. Codon positions included were 1st+2nd+3rd+Noncoding. All ambiguous positions were removed for each sequence pair (pairwise deletion option). There were a total of 29904 positions in the final dataset. Evolutionary analyses were conducted in MEGA X [4].

Structural mapping

Structural mapping of mutations in the spike glycoprotein between SARS and the current SARS-CoV-2 was done using MAFFT [3] for sequence alignment and a custom Perl script to map differences to the known crystal structure of SARS spike glycoprotein (PDB:6ACG [5]) using YASARA [6].

1. Shu, Y.; McCauley, J. GISAID: Global initiative on sharing all influenza data - from vision to reality. *Euro Surveill.* **2017**, *22*.
2. Elbe, S.; Buckland-Merrett, G. Data, disease and diplomacy: GISAID's innovative contribution to global health. *Glob Chall* **2017**, *1*, 33–46.
3. Nakamura, T.; Yamada, K.D.; Tomii, K.; Katoh, K. Parallelization of MAFFT for large-scale multiple sequence alignments. *Bioinformatics* **2018**, *34*, 2490–2492.
4. Kumar, S.; Stecher, G.; Li, M.; Knyaz, C.; Tamura, K. MEGA X: Molecular Evolutionary Genetics Analysis across Computing Platforms. *Mol. Biol. Evol.* **2018**, *35*, 1547–1549.
5. Song, W.; Gui, M.; Wang, X.; Xiang, Y. Cryo-EM structure of the SARS coronavirus spike glycoprotein in complex with its host cell receptor ACE2. *PLoS Pathog.* **2018**, *14*, e1007236.
6. Krieger, E.; Vriend, G. YASARA View - molecular graphics for all devices - from smartphones to workstations. *Bioinformatics* **2014**, *30*, 2981–2982.
